# Helping first-time mothers establish and maintain breastfeeding: Access to someone who can provide breastfeeding advice is an important factor

**DOI:** 10.1371/journal.pone.0287023

**Published:** 2023-06-28

**Authors:** Brittany A. Massare, Nicole M. Hackman, Kristin K. Sznajder, Kristen H. Kjerulff

**Affiliations:** 1 Department of Pediatrics, Penn State College of Medicine, Hershey, Pennsylvania, United States of America; 2 Departments of Public Health Sciences, Penn State College of Medicine, Hershey, Pennsylvania, United States of America; 3 Departments of Public Health Sciences and Obstetrics and Gynecology, Penn State College of Medicine, Hershey, Pennsylvania, United States of America; University of North Carolina at Chapel Hill Gillings School of Global Public Health, UNITED STATES

## Abstract

**Background and aims:**

First-time mothers often need help with breastfeeding and may feel isolated and uncertain about whom they can turn to for help with breastfeeding challenges. Exploration of whether access to breastfeeding advice helps new mothers initiate and continue breastfeeding is necessary. This study investigated the associations between ease of access to breastfeeding advice for first-time mothers and breastfeeding initiation and duration.

**Methods:**

This was a prospective, longitudinal cohort study of 3,006 women who delivered their first child in Pennsylvania, USA; with prenatal and postpartum interviews. At 1-month postpartum participants reported the extent to which they had access to “Someone to give you advice about breastfeeding if you needed it”, via a 5-point scale ranging from “none of the time” to “all of the time”.

**Results:**

There were 132 women (4.4%) who reported that they had access to someone to give them advice about breastfeeding “none of the time”; 697 (23.3%) reported access “a little of the time” or “some of the time”; and 2,167 (72.3%) reported access “most of the time” or “all of the time”. While the majority of the new mothers were breastfeeding at 1-month postpartum (72.5%), less than half were still breastfeeding at 6-months postpartum (44.5%). The higher the level of access to advice about breastfeeding the more likely women were to establish breastfeeding by 1-month postpartum and to still be breastfeeding at 6-months.

**Conclusions:**

For first-time mothers, ease of access to someone who can give them advice about breastfeeding facilitates breastfeeding establishment and continuation.

## Introduction

The health advantages for breastfeeding to mothers and their infants are well documented [1]. Breastfed infants are less likely to develop acute infections, chronic disease such as asthma, and are more likely to have stronger socioemotional and developmental outcomes [2]. Women who breastfeed have reduced rates of breast cancer and ovarian cancer, type 2 diabetes mellitus [1] and may even have decreased rates of postpartum depression [3]. The American Academy of Pediatrics (AAP) and the World Health Organization (WHO) both recommend exclusive breastfeeding of infants through 6 months of age and continued breastfeeding through at least the first year of life [4, 5].

According to the WHO, 44% of infants are exclusively breastfed at 6 months of age [4]. The WHO goal for 2030 is for 70% of infants to be exclusively breastfed at 6 months of age [4]. To meet the WHO 2030 target, we need to take a critical look at understudied variables in breastfeeding initiation and duration to identify novel interventions to promote continued breastfeeding. Social support such as from healthcare professionals, support groups, female relatives, and fathers of the infant can each impact breastfeeding initiation and duration [6–8]. Kummer et al [9] found that breastfeeding initiation was significantly higher when parents identified an emotional support person. Women who were advised to breastfeed by either a medical provider or a counselor at the Special Supplemental Nutrition Program for Women, Infants and Children (WIC) were more likely to breastfeed, whereas women who were advised to bottle-feed by a WIC counselor were less likely to breastfeed [10]. Among 48.7% of Italian mothers who encountered breastfeeding difficulties, twenty percent solved the difficulty themselves, whereas 11.6% reported engaging a friend or relative for help. More than half of the women who did not have their difficulty solved stopped breastfeeding [11]. These previous studies illustrate that a medical professional, such as an International Board-Certified Lactation Consultant (IBCLC), or a strong support person who can provide breastfeeding advice, are important variables that can increase the rate of breastfeeding to at least 6 months. More research is needed to understand the role of breastfeeding advice in maternal decisions surrounding breastfeeding, how to overcome common breastfeeding challenges, and how to increase duration of breastfeeding. Using data from a large-scale, prospective interview study, we investigated the associations between ease of access to breastfeeding advice for first-time mothers and breastfeeding establishment and duration.

## Materials and methods

### Research design

The First Baby Study (FBS) is a prospective, longitudinal cohort study [12, 13]. In this study, pregnant primiparous women aged 18 to 35 years old were recruited from childbirth education classes, hospital tours, intranet-postings, advertisements and targeted mailings throughout the state of Pennsylvania. Women were recruited in 2009 to 2011 and followed for a three-year period via telephone interviews. The primary purpose of the FBS was to investigate the relationship between mode of first delivery and subsequent childbearing. A planned secondary objective was to investigate factors associated with breastfeeding establishment and duration in this cohort. Participants were interviewed by telephone during the third trimester of pregnancy and at 7 time points after childbirth: 1, 6, 12, 18, 24, 30 and 36 months postpartum.

### Setting and relevant context

The study participants were residents of Pennsylvania, a state in the northeastern United States, who delivered at 78 hospitals located throughout the state. The participants were more likely to have a college degree, to be married and to be white-non-Hispanic than women having their first birth in the state of Pennsylvania as a whole, as we have reported previously [12].

### Sample

The inclusion criteria were age 18–35 years old at the time of study recruitment, primiparous, English or Spanish speaking, and planning to deliver in a hospital in Pennsylvania. Exclusion criteria were a surrogate pregnancy, planning for the child to be adopted, a previous pregnancy of 20 weeks gestation or longer, a previous cesarean delivery, and delivering before 34 weeks gestation. There were 3,006 women who completed the baseline and 1-month postpartum interviews. Birth certificate and hospital discharge data for these 3,006 study participants were obtained and matched to the interview data by the Pennsylvania Health Care Cost Containment Council. Among these 3,006 women, 10 answered “don’t know” to the question on advice about breastfeeding and were excluded, leaving a sample size of 2,996 for investigation of the association between access to advice about breastfeeding and establishment of breastfeeding (defined as breastfeeding at 1-month postpartum). Duration of breastfeeding was assessed based on women’s answers to questions about breastfeeding at the 1 and 6-month interviews. There were 49 women for whom we were unable to determine breastfeeding duration because they did not participate in the 6-month interview, leaving a sample size of 2,947 women for analysis of breastfeeding duration. The FBS was approved by the Penn State College of Medicine Institutional Review Board (IRB), as well as all other IRBs involved with participant recruitment. This included the IRBs associated with low-income clinics and hospitals located throughout the state of Pennsylvania. All participants completed signed, informed consent.

### Data collection

Data for this study of breastfeeding outcomes were obtained from telephone interviews conducted during pregnancy (baseline), and at 1 and 6 months postpartum, as well as birth certificate and hospital discharge data. Women were interviewed by telephone by trained professional interviewers employed by the Penn State Center for Survey Research. The interviewers participated in training classes conducted by the study investigators which covered a variety of topics including the purpose of each interview question, the meaning of specific medical terms, how to ask questions in a sensitive and non-judgmental manner, and how to record answers to open-ended questions.

### Measurement

Demographic and background information were obtained in the baseline interview and verified in relation to the birth certificate and hospital discharge data. We used the official U.S. Census Bureau levels for poverty to determine poverty level for each participant. Participants were categorized as living in poverty if their family income was ≤ 100% of the federal poverty level (FPL), near poverty if their family income was 101%-200% of the FPL, and not living in poverty if their family income was above 200% of the FPL [14]. Pre-pregnancy body mass index (BMI) was calculated as weight in kilograms divided by height in meters squared [15]. Women were asked “During your current pregnancy have you smoked cigarettes every day, some days or not at all?” Women who answered “every day” or “some days” were classified as smoking during the pregnancy. Social support during pregnancy was measured using a 5-item version of the Medical Outcome Study (MOS) Social Support Scale [16]. The Cronbach’s alpha for this scale was 0.88. Total scores could range from 5 to 25, with higher scores indicating higher levels of social support. Total scores were classified into three categories of social support: low (5–19), medium (20–23) and high (24–25). In the baseline interview women were asked about their plans for breastfeeding their newborn and returning to work after childbirth. In the 1-month postpartum interview women were asked if they had attended any education classes during the pregnancy, covering such topics as childbirth, breastfeeding, or how to take care of a baby. If they answered “yes” they were then asked what topics were covered in these classes. Mode of delivery was measured by maternal self-report and verified in relation to the hospital discharge data. Gestational age was obtained from the birth certificate data. In the 1-month interview, women were asked about various types of support available to them since they had their baby, including “Someone to give you advice about breastfeeding if you needed it”. They responded via a 5-point scale: “None of the time”, “A little of the time”, “Some of the time”, “Most of the time” and “All of the time”. Answers to this question were classified into three categories:1.) “none of the time”; 2.) “a little of the time” or “some of the time”; and 3.) “most of the time” or “all of the time”. At the 1-month interview women were asked “Have you ever breastfed or tried to breastfeed your baby?”. If they said “yes” they were then asked “Are you still breastfeeding your baby?” If they said “no” they were then asked how long they breastfed and why they were no longer breastfeeding. Three categories of breastfeeding establishment were assessed: 1.) the mother did not try to breastfeed, 2.) the mother tried to breastfeed but quit within the first month postpartum, and 3.) the mother was breastfeeding at 1-month postpartum (breastfeeding established). Women who had reported that they were breastfeeding at the 1-month interview were asked at the 6-month interview if they were still breastfeeding. To measure breastfeeding duration women were classified into three categories: 1.) breastfed for less than 1-month (including those who did not initiate breastfeeding); 2.) breastfeeding at 1-month, but no longer breastfeeding at 6-months; and 3.) breastfeeding at 6-months.

### Data analysis

Frequencies were calculated for the independent variable (degree of access to advice about breastfeeding), the demographic and background factors (potential confounders) and the dependent variables (breastfeeding establishment and duration). Chi-square analyses were conducted to determine the associations between the confounder variables and the independent variable, as well as the associations between the independent variable and the two dependent variables (breastfeeding establishment and breastfeeding duration). We measured the average number of days of breastfeeding among those who initiated breastfeeding but were no longer breastfeeding at 1-month postpartum. Women’s answers to the open-ended question about why they were no longer breastfeeding were coded into categories. Two multivariable logistic regression models were developed to measure the associations between the degree of access to advice about breastfeeding and the two dependent variables (breastfeeding establishment and breastfeeding duration), controlling for the variables that were significantly associated with access to advice about breastfeeding in the bivariate analysis, including maternal age, education, race/ethnicity, poverty, pre-pregnancy BMI, smoking during pregnancy, social support during pregnancy, plans to return to work, attending breastfeeding class during pregnancy, and gestational age. All variables were entered into these two models at the same time. The breastfeeding establishment model compared women who reported that they were breastfeeding at the 1-month interview to those who were not, and the breastfeeding duration model compared women who were breastfeeding at 6-months postpartum to those who were not.

## Results

Demographic and background characteristics of the study population can be seen in Table 1. More than half of the participants had college degrees or higher levels of education (56.7%), and the majority were married (70.5%), white non-Hispanic (83.3%), and not living in poverty (80.0%). Most reported during pregnancy that they planned to breastfeed (92.4%), but less than half attended a breastfeeding class during the pregnancy (41.6%). Breastfeeding classes were generally in-person and provided by lactation specialists at the hospitals where women planned to deliver. A small percent of women (4.4%) reported that they had access to someone to give them advice about breastfeeding none of the time, and the majority of the study participants reported access most or all of the time (72.3%). Women who reported that they had access to advice about breastfeeding none of the time were more likely to be in the youngest age group, in the lowest education group, and unmarried. In addition, these women were more likely to be living in poverty or near poverty, to be overweight or obese, and to have smoked during pregnancy. These women were less likely to be planning to breastfeed (72.0%) than the women with higher levels of access to breastfeeding advice [94.5% of women with access a little or some of the time and 92.9% of those with access most or all of the time, p < .001] and were less likely to have taken a breastfeeding class during pregnancy (21.2%) in comparison to the women with higher levels of access to breastfeeding advice [45.3% of women with access a little or some of the time and 41.6% of those with access most or all of the time, p< .001]. Women who reported access to someone to give them advice about breastfeeding most or all of the time were considerably more likely to report high levels of social support during pregnancy (47.9%) than women who reported access to advice about breastfeeding a little or some of the time (26.6%) or none of the time (28.0%), p < .001. Mode of delivery was not associated with degree of access to breastfeeding advice, but gestational age was associated, such that the women who reported high levels of access were less likely to have delivered preterm than the women with lower access to advice about breastfeeding.

**Table 1:** Demographic and background factors by access to breastfeeding advice among first-time mothers (N = 2,996).

Characteristic	Overall	Access to Advice about Breastfeeding			p-Value
		132 (4.4%)	697 (23.3%)	2,167 (72.3%)	
		None of the time	A little or some of the time	Most or all of the time	
	n (%)	n (%)	n (%)	n (%)	
Age					< .001
18–24	806 (26.9)	63 (47.7)	175 (25.1)	568 (26.2)	
25–29	1,190 (39.7)	41 (31.1)	274 (39.3)	875 (40.4)	
30–35	1,000 (33.4)	28 (21.2)	248 (35.6)	724 (33.4)	
Education					< .001
High school or less	496 (16.6)	33 (25.0)	101 (14.5)	362 (16.7)	
Some college/technical	801 (26.7)	53 (40.2)	177 (25.4)	571 (26.3)	
College degree+	1,699 (56.7)	46 (34.8)	419 (60.1)	1,234 (56.9)	
Marital status					< .001
Married	2,113 (70.5)	58 (43.9)	500 (71.7)	1,555 (71.8)	
Not married	883 (29.5)	74 (56.1)	197 (28.3)	612 (28.2)	
Race/ethnicity					.023
White non-Hispanic	2,497 (83.3)	95 (72.0)	579 (83.1)	1,823 (84.1)	
Black non-Hispanic	219 (7.3)	17 (12.9)	53 (7.6)	149 (6.9)	
Hispanic	164 (5.5)	13 (9.8)	35 (5.0)	116 (5.4)	
Other	116 (3.9)	7 (5.3)	30 (4.3)	79 (3.6)	
Poverty status^a^					< .001
Poverty	253 (8.4)	26 (19.7)	50 (7.2)	177 (8.2)	
Near poverty	345 (11.5)	26 (19.7)	77 (11.0)	242 (11.2)	
Not poverty	2,398 (80.0)	80 (60.6)	570 (81.8)	1,748 (80.7)	
Pre-pregnancy BMI^b^					.019
< 25.0	1,709 (57.1)	58 (43.9)	387 (55.5)	1,264 (58.4)	
25.0–29.9	666 (22.2)	39 (29.5)	164 (23.5)	463 (21.4)	
30+	619 (20.7)	35 (26.5)	146 (20.9)	438 (20.2)	
Smoked in pregnancy					< .001
Yes	312 (10.4)	30 (22.7)	72 (10.3)	210 (9.7)	
No	2,684 (89.6)	102 (77.3)	625 (89.7)	1,957 (90.3)	
Social support in pregnancy					< .001
Low (5–19)	506 (16.9)	48 (36.4)	188 (27.0)	270 (12.5)	
Medium (20–23)	1,227(41.0)	47 (35.6)	323 (46.4)	857 (39.6)	
High (24–25)	1,260 (42.1)	37 (28.0)	185 (26.6)	1,038 (47.9)	
Plan to breastfeed					< .001
Yes	2,767 (92.4)	95 (72.0)	659 (94.5)	2,013(92.9)	
No or don’t know	229 (7.6)	37 (28.0)	38 (5.5)	154 (7.1)	
Plans to return to work^c^					.026
< 2 months	1,274 (42.5)	42 (31.8)	310(44.5)	922 (42.5)	
≥ 2 months or no plans	1,722 (57.5)	90 (68.2)	387 (55.5)	1,245 (57.5)	
Breastfeeding class					< .001
Yes	1,245 (41.6)	28 (21.2)	316 (45.3)	901 (41.6)	
No	1,751 (58.4)	104 (78.8)	381 (54.7)	1,266 (58.4)	
Mode of delivery					.109
Vaginal	2,135 (71.3)	87 (65.9)	482 (69.2)	1,566 (72.3)	
Cesarean	861 (28.7)	45 (34.1)	215 (30.8)	601 (27.7)	
Gestational age (wks)					.010
Preterm (34–36)	120 (4.0)	8 (6.1)	40 (5.7)	72 (3.3)	
Early term (37–38)	572 (19.1)	29 (22.0)	136 (19.5)	407 (18.8)	
Full term (39–40)	1,808 (60.3)	77 (58.3)	389 (55.8)	1,342 (61.9)	
Late/post term (41+)	496 (16.6)	18 (13.6)	132 (18.9)	346 (16.0)	

Column percents shown, p-values resulting from chi-square analyses

^a^Poverty = family income (FI) ≤ 100% federal poverty level (FPL); Near poverty = FI 101–200% FPL; Not poverty = FI > 200% FPL.

^b^BMI = body mass index, calculated as weight in kilograms divided by height in meters squared.

^c^Reported by mother during third trimester of pregnancy.

Overall, 8.4% of the women did not try to breastfeed, 19.2% tried but quit within the first month, and 72.5% were breastfeeding at 1-month postpartum (Table 2). Access to advice about breastfeeding was strongly associated with breastfeeding establishment. A third of the women (33.3%) who reported access to breastfeeding advice “none of the time” did not try to breastfeed and an additional third (34.8%) tried but quit within the first month. Only 31.8% of women without access to breastfeeding advice were still breastfeeding at 1-month postpartum, in comparison to 70.7% of the women who reported access to advice about breastfeeding “a little” or “some of the time”, and 75.5% among those who reported access to advice about breastfeeding “most” or “all of the time” (p < .001). Women who reported that they had tried to breastfeed but were no longer breastfeeding by 1-month postpartum had breastfed for a mean (standard deviation) of 11.9 (7.9) days. The most common reasons they were no longer breastfeeding included the following: not enough milk (34.0%), the baby would not latch (27.3%), and it was too difficult (23.9%).

**Table 2:** Access to advice about breastfeeding among first-time mothers by breastfeeding establishment (N = 2,996).

	Category of Breastfeeding Establishment			p-Value
	Did not try	Tried but quit in 1st month	Breastfeeding at 1-month	
	249 (8.4%)	575 (19.2%)	2,172 (72.5%)	
	n (%)	n (%)	n (%)	
Access to advice about breastfeeding				< .001
None of the time	44 (33.3)	46 (34.8)	42 (31.8)	
A little or some of the time	37 (5.3)	167 (24.0)	493 (70.7)	
Most or all of the time	168 (7.8)	362 (16.7)	1,637 (75.5)	

Row percents shown, p-values resulting from chi-square analysis

Out of the total women represented in our analysis, 28.0% were not breastfeeding by 1 month postpartum, 27.5% were breastfeeding at 1 month but not at 6 months, and 44.5% were still breastfeeding at 6 months postpartum (Table 3). Access to advice about breastfeeding was also strongly associated with breastfeeding duration. Only 14.6% of the women who reported access to advice about breastfeeding “none of the time” were still breastfeeding at 6-months postpartum, in comparison to 38.6% of the women who reported access to breastfeeding advice “a little” or “some of the time”, and 48.2% of the women who reported access to breastfeeding advice “most” or “all of the time” (p < .001).

**Table 3:** Access to advice about breastfeeding among first-time mothers by breastfeeding duration (N = 2,947).

	Breastfeeding Duration			p-Value
	< 1 month	1 to < 6 months	≥ 6 months	
	824 (28.0%)	811 (27.5%)	1,312 (44.5)	
Access to advice about breastfeeding				< .001
None of the time	90 (69.2)	21 (16.2)	19 (14.6)	
A little or some of the time	204 (29.7)	217 (31.6)	265 (38.6)	
Most or all of the time	530 (24.9)	573 (26.9)	1,028 (48.2)	

Row percents shown, p-value resulting from chi-square analysis

In the multivariable logistic regression model to measure the association between access to advice about breastfeeding and the outcome of breastfeeding establishment (Table 4), women who reported access to someone to give them advice about breastfeeding “a little” or “some of the time” were substantially more likely to establish breastfeeding as of 1 month postpartum than those who reported access to advice about breastfeeding “none of the time” (OR 3.45, 95% CI 2.21–5.39, p < .001), with similar results when comparing women who reported access “most” or “all of the time” to those who reported access “none of the time” (OR 5.37, 95% CI 3.50–8.23, p < .001). For the outcome of breastfeeding duration ≥ 6 months (Table 4), women who reported access to advice about breastfeeding “a little” or “some of the time” were significantly more likely to breastfeed until at least 6 months compared with women who reported access to advice about breastfeeding “none of the time” (OR 2.28, 95% CI 1.31–3.95, p = .004). Women who reported access to advice about breastfeeding “most” or “all of the time” were also significantly more likely to still be breastfeeding 6 months postpartum in comparison to those who reported access “none of the time” (OR 4.04, 95% CI 2.36–6.90, p < .001).

**Table 4:** Fully adjusted multivariable logistic regression models of the association between access to advice about breastfeeding and breastfeeding establishment and duration.

	Breastfeeding established		Breastfeeding duration ≥ 6 months	
	Adjusted OR (95% CI)	p-Value	Adjusted OR (95% CI)	p-Value
Access to advice about breastfeeding				
None of the time	Reference		Reference	
A little or some of the time	3.45 (2.21–5.39)	< .001	2.28 (1.31–3.95)	.004
Most or all of the time	5.37 (3.50–8.23)	< .001	4.04 (2.36–6.90)	< .001

Both models adjusted for maternal age, education, race/ethnicity, poverty, pre-pregnancy BMI, smoking during pregnancy, social support during pregnancy, plans to return to work, attending breastfeeding class during pregnancy and gestational age.

## Discussion

Our analysis indicates that ease of access to someone who can provide advice about breastfeeding for first-time mothers is strongly associated with breastfeeding establishment and continuation to at least the age of 6 months. We found that younger, unmarried women with a high school degree or less, a group typically at risk for reduced breastfeeding initiation and duration, were more likely to have limited or no access to someone who could provide breastfeeding advice if needed. We add novel evidence about the role that ease of access to advice about breastfeeding plays in breastfeeding establishment and duration.

The 2030 target set by WHO challenges health care professionals to help 70% of babies be exclusively breastfed until 6 months of age [4]. We need to explore novel methods to meet this challenge. This goal, along with the AAP’s recommendation [5] to exclusively breastfeed until 6 months of age, was put in place to help infants and mothers thrive from the known benefits of breastfeeding. Women who reported that they had access to someone who could give them advice about breastfeeding “none of the time” were more likely to report that they did not try to breastfeed than women with higher levels of access to advice about breastfeeding. If women without access to advice did try to breastfeed, they were more likely to stop breastfeeding within the first month. The two most common reasons these women reported for early breastfeeding cessation were ‘not enough milk’ and ‘the baby would not latch’. These are common challenges that women face when beginning breastfeeding [17]. With proper advice and support, it is possible these challenges can be overcome and breastfeeding continued. In addition, women with access to advice about breastfeeding “none of the time” were substantially less likely to still be breastfeeding at 6 months postpartum than those with higher levels of access to breastfeeding advice. The higher the degree of access to advice about breastfeeding, the more likely women were to still be breastfeeding at 6 months postpartum. These results underline the importance of access to breastfeeding advice to help women establish and continue breastfeeding their newborns.

Access to an IBCLC increases breastfeeding rates [18]. Cohen et al. [19] in a review of the literature identified breastfeeding education, through prenatal breastfeeding classes, lactation consultation before or after delivery, and counselling on breastfeeding, as a factor positively associated with breastfeeding initiation and continuation. Schindler-Ruwisch et al. [20] reported that after reviewing numerous mobile device breastfeeding support resources, including text-messaging and mobile applications, maternal breastfeeding support from peers and professionals positively affects breastfeeding rates. Additionally, the effect of social media breastfeeding support groups, comprised of peers and experienced breastfeeding mothers, was evaluated by Wilson in 2020 [21]. In this study, mothers who actively participated in these support groups were more likely to exclusively breastfeed at 6 months postpartum.

‘Breastfeeding support’ is a broad term that encompasses a range of elements including encouragement, reassurance, peer modeling, information, and advice [22–24]. In this study we focused specifically on one aspect of breastfeeding support—breastfeeding advice—because previous studies have found that many women who cease breastfeeding early report breastfeeding difficulties, such as perceiving that they do not have enough milk or the baby does not latch properly [11, 25]. These are breastfeeding problems that can often be ameliorated if the new mother has access to practical advice, such as how to position the baby while feeding, and how often to breastfeed [22, 26, 27]. In our study we found that ease of access to someone who could give first-time mothers advice about breastfeeding was associated with both establishment of breastfeeding and maintenance of breastfeeding, even after controlling for demographic factors, general social support and attendance at a breastfeeding class during pregnancy. In addition, the effect of ease of access was linearly associated such that the easier the access the more likely women were to establish and maintain breastfeeding. If a woman reports that she has access to someone who can give her advice about breastfeeding “most of the time” or “all of the time”, it seems likely that this person would be someone who has breastfeeding experience and is a friend or family member that the new mother would feel comfortable reaching out to if and when needed, even at 2 AM.

Access to advice can help women initiate breastfeeding and prolong breastfeeding duration [7, 17, 21]. Creating a breastfeeding support plan for each new mother can capitalize on insights about access to advice for breastfeeding and methods of sharing that advice could further improve breastfeeding establishment and duration. Shwarz et al. [28] have created a table of tips to help new mothers with breastfeeding that includes encouraging mothers during prenatal counseling to develop a breastfeeding support plan. Smith et al. [29] concluded that advice from multiple sources had a cumulative effect on improving breastfeeding rates. Additionally, Hernandez-Cordero et al. [30] concluded that even though it is known that breastfeeding support is necessary, more sustainable and effective large-scale breastfeeding programs need to be available. The WHO states that hospital providers should “coordinate discharge so that parents and their infants have timely access to ongoing support and care [31]. We recommend planning the method to access breastfeeding advice proactively, such as during a prenatal visit or postpartum hospital stay. In conjunction with the new mother, providers can help empower women to create a list of ways to obtain breastfeeding advice should it be needed. The personalized support plan could include family members, friends, community groups, IBCLCs, mobile apps, or social media support groups.

### Limitations

Although nearly 3,000 first-time mothers in Pennsylvania were included in the study, participants were more likely to be white non-Hispanic, married, and have a college degree and private insurance, which is consistent with other participation studies, but could affect the generalizability of the findings [32, 33]. Breastfeeding establishment and continuation information was collected via a telephone survey, possibly leading to increased reports of breastfeeding due to mothers often knowing this is the desired feeding method by medical providers. In addition, our survey asked about access to advice related to breastfeeding, but did not expand upon what type of advice was available, or from whom. Future studies could expand on this knowledge and identify the forms of advice that are most successful at helping mothers to breastfeed.

## Conclusions

In this study we found that accessibility of breastfeeding advice plays an important role in breastfeeding establishment and duration. We suggest that improving breastfeeding support and frequency of access to advice from friends, family, IBCLCs, community members, and social media resources should be a focus of future breastfeeding initiatives.
